# Impact of smoking on dendritic cell phenotypes in the airway lumen of patients with COPD

**DOI:** 10.1186/1465-9921-15-48

**Published:** 2014-04-18

**Authors:** Paul Stoll, Ann-Sophie Heinz, Kai Bratke, Andrea Bier, Katharina Garbe, Michael Kuepper, J Christian Virchow, Marek Lommatzsch

**Affiliations:** 1 Department of Pneumology and Critical Care Medicine, University of Rostock, Ernst-Heydemann-Str. 6, 18057 Rostock, Germany

**Keywords:** Dendritic cells, COPD, Smoking, Airway

## Abstract

**Background:**

Myeloid dendritic cells (DCs) are increased in the airway wall of patients with chronic obstructive pulmonary disease (COPD), and postulated to play a crucial role in COPD. However, DC phenotypes in COPD are poorly understood.

**Methods:**

Function-associated surface molecules on bronchoalveolar lavage fluid (BALF) DCs were analyzed using flow cytometry in current smokers with COPD, in former smokers with COPD and in never-smoking controls.

**Results:**

Myeloid DCs of current smokers with COPD displayed a significantly increased expression of receptors for antigen recognition such as BDCA-1 or Langerin, as compared with never-smoking controls. In contrast, former smokers with COPD displayed a significantly decreased expression of these receptors, as compared with never-smoking controls. A significantly reduced expression of the maturation marker CD83 on myeloid DCs was found in current smokers with COPD, but not in former smokers with COPD. The chemokine receptor CCR5 on myeloid DCs, which is also important for the uptake and procession of microbial antigens, was strongly reduced in all patients with COPD, independently of the smoking status.

**Conclusion:**

COPD is characterized by a strongly reduced CCR5 expression on myeloid DCs in the airway lumen, which might hamper DC interactions with microbial antigens. Further studies are needed to better understand the role of CCR5 in the pathophysiology and microbiology of COPD.

## Introduction

An abnormal immune response to inhaled noxious agents such as tobacco smoke is a key pathogenetic feature of chronic obstructive pulmonary disease (COPD) [1]. The immunological dysfunction in COPD leads to a pathologic formation of lymphoid follicles around the airways and contributes to small airway obstruction [2]. Dendritic cells (DCs), which are subdivided into myeloid DCs (mDCs) and plasmacytoid DCs (pDCs), are specialized antigen-presenting cells which initiate and control adaptive immune responses in the lung. These cells were postulated to play a key role in the formation of lymphoid follicles in small airways of patients with COPD [3-6]. In line with this postulate, histologic studies using lung tissue from patients with COPD showed that COPD is characterized by increased numbers of Langerin expressing DCs in the epithelium and the adventitia of small airways and changes in selected costimulatory molecules [7-11]. A further analysis of the phenotype and function of these DCs in human lung tissues was hampered by technical limitations of immunohistochemistry (allowing to detect only a few markers on one cell). For instance, it is currently unclear whether the decrease in CD83 positive cells in small airways of patients with COPD represents a decrease in the total number of DCs or a reduced expression of this maturation marker on DCs [12].

Over the last years, we have established a flow cytometric method to comprehensively analyze function-associated surface molecules on DCs in human bronchoalveolar lavage fluid (BALF) [13-16]. Using this method, we have demonstrated that BALF mDCs of smokers with normal spirometry display a strong upregulation of specific receptors for antigen recognition and presentation such as CD1a and CD1c (BDCA-1) [17], Langerin (CD207) [18], Macrophage Mannose Receptor (CD206) [19], BDCA-4 (CD304, Neuropilin-1) [20], CD80 and CD86 [21], suggesting that these changes represent a physiologic adaptation of airway mDCs to cigarette smoke exposure [14]. On the other hand, some surface molecules on BALF mDCs were unchanged in smokers, including BDCA-3 (CD141, Thrombomodulin [22]) and CD83 (a maturation marker of DCs [23]) [14]. However, there is currently little information on DC phenotypes in patients with COPD. In addition, the relationship of these phenotypes to the smoking status of patients with COPD is poorly understood. It was the aim of this clinical study, therefore, to analyze function-associated surface molecules of BALF DCs in COPD for the first time, and to relate these findings to the current smoking status of the patients.

## Methods

### Subjects

Controls were recruited using public notices in Rostock (Germany). Patients were recruited at the University Hospital of Rostock (Germany). Inclusion criteria for patients with COPD were as follows: 1. age between 35 and 75 years, 2. smoking history of at least 10 pack years, 3. a ratio of FEV_1_/FVC of < 70% after inhalation of a short-acting beta-agonist. Current smokers with COPD were defined as subjects smoking at least 10 cigarettes per day, former smokers were defined as subjects who quit smoking at least one year ago. Controls were recruited using the following inclusion criteria: 1. age between 35 and 75 years, 2. no history of smoking and no exposure to smoking partners or relatives at home, 3. no history of any chronic lung disease. For both groups, exclusion criteria were as follows: 1. any history of malignant or chronic inflammatory diseases, 2. any signs of a respiratory tract infection within the last 2 weeks prior to bronchoscopy, 3. a FEV_1_ of < 30% of the predicted value. The study was approved by the local ethics committee of Ärztekammer Mecklenburg-Vorpommern, Rostock (Germany). All participants gave their written informed consent.

### Study design

All subjects were examined between 8 and 11 am. Lung function tests and bronchoscopy were performed on the same day. In a first step, informed consent was obtained and a structured history was taken. Subsequently, body plethysmography was performed and the diffusion capacity (DLCO) measured (Masterscreen, Jaeger, Carefusion, Hoechberg, Germany). Then, 10 ml of blood were taken for laboratory analyses and for the quantification of plasmacytoid and myeloid DCs in peripheral blood. Finally, a bronchoscopy was performed, with an inhalation of 4% lidocaine for 15 minutes prior to the procedure (Pari-Boy, Starnberg, Germany).

### Bronchoalveolar lavage and flow cytometry

**Figure 1:**
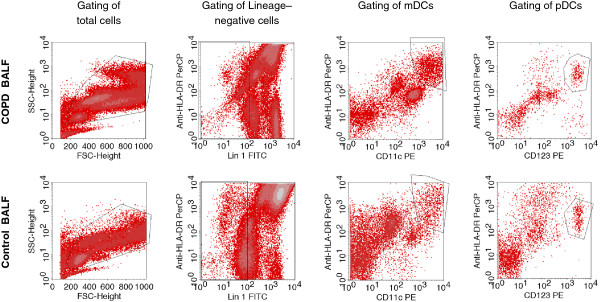
**Gating of Dendritic cells in BALF.** Total cells were gated in a FSC/SSC-plot (*first column*). In this cell population, lineage negative/dim (lin^neg/dim^) cells were gated (*second column*). In the lin^neg/dim^ gate, cells expressing HLA-DR and CD11c were identified as mDCs (lin^neg/dim^HLA-DR^+^CD11c^+^)(*third column*), and cells expressing HLA-DR and CD123 were identified as pDCs (lin^neg/dim^HLA-DR^+^CD123^+^)(*fourth column*). The figure shows the BALF from a patient with COPD (*upper panel*) and a healthy never-smoker (*lower panel*).

**Figure 2:**
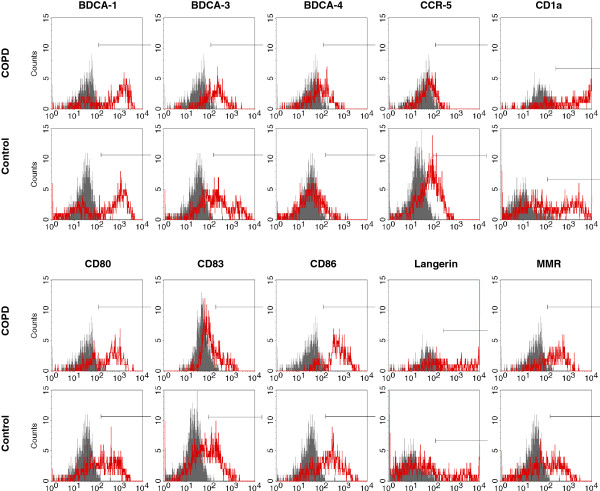
**Histogram plots of mDC surface markers in BALF.** Myeloid DCs were identified as lin^neg/dim^HLA-DR^+^CD11c^+^ cells. Histograms show the staining of these cells with antibodies against surface markers (marker antibody, *red*) compared to the staining with respective isotype control antibodies (control antibody, *grey*). The figure shows examples from a current smoker with COPD (COPD) and a healthy never-smoker (Control).

Bronchoalveolar lavage was performed using flexible bronchoscopes (Olympus, Hamburg, Germany) as described [13-16]. Briefly, the bronchoscope was wedged into a subsegment of the right middle lobe and a total of 100 ml prewarmed sterile saline was instilled. The fluid was recovered by gentle aspiration. BALF cells were isolated, counted and then analysed with four-colour flow cytometry as previously described [13-16], using the antibodies detailed in Additional file 1: Table S1. Blood dendritic cells were analyzed using freshly collected EDTA-blood as described [24]. Among cells negative/dim for lineage markers (CD3, CD14, CD16, CD19, CD20, CD56) in BALF or peripheral blood, mDCs were defined as CD11c^+^HLA-DR^+^lin^neg/dim^ cells and pDCs as CD123^+^HLA-DR^+^lin^neg/dim^ cells [13-16](Figure 1). The inclusion of cells weakly positive for lineage markers (“lineage dim cells”) was chosen because DCs can express low levels of lineage markers such as CD14 [25]. This approach ensures that most of these DCs are included in the analysis. However, we cannot completely exclude the possibility that some of these DCs were omitted by our gating strategy because there is a smooth transition from monocytes to myeloid DCs regarding the expression of surface molecules such as CD14 in human BALF. Surface molecule expression on CD11c^+^HLA-DR^+^lin^neg/dim^ cells (mDCs) was quantified in histogram plots using isotype control antibodies to discriminate between specific and non-specific staining (Figure 2). The ligands of the chemokine receptor CCR5 CCL3 (MIP-1alpha), CCL4 (MIP-1beta) and CCL5 (RANTES) (detection limits: 4 pg/ml) were measured in BALF supernatants using commercial ELISA Kits as described by the manufacturer (R&D Systems, Wiesbaden, Germany).

### Statistical analysis

Statistical analysis was performed using SPSS Statistics (SPSS Inc., Chicago, Illinois, USA). The majority of parameters was not normally distributed. Therefore, parameters were expressed as medians (minimum – maximum). For the comparison of groups, the Mann-Whitney-*U* test for unrelated samples was used. Correlation analyses were performed using Spearman’s correlation coefficient. Probability values of p < 0.05 were regarded as significant.

## Results

### Subject characteristics

Ten never-smokers, 11 former smokers with the diagnosis of COPD and 13 current smokers with the diagnosis of COPD were included in the study based on the inclusion and exclusion criteria. Subject characteristics are detailed in Table 1. There were no differences in age and body height between the groups. Former smokers displayed a higher body weight than current smokers with COPD (Table 1). There was no difference in the smoking history (pack years), lung function parameters or the diffusion capacity between current smokers and former smokers with COPD (Table 1). All patients treated with an ICS were also treated with a LABA, in a fixed ICS/LABA combination in one inhaler (n = 10). The other patients were treated with a LAMA or a LABA or a combination of both, or with a rescue medication only (n = 14).

**Table 1:** Subject characteristics

	**Control group (C) n = 10**	**COPD total group (T) n = 24**	**COPD current smoker (S) n = 13**	**COPD former smoker (F) n = 11**	**C-T p**	**C-S p**	**C-F p**	**S-F p**
**Age** (years)	51.0 [40…68]	55.0 [35…73]	52.0 [35…63]	62.0 [38…73]	0.491	0.879	0.132	0.055
**Gender** (m / f)	6 / 4	18 / 6	10 / 3	8 / 3	NA	NA	NA	NA
**BMI** (kg*m^−2^)	25.6 [23.2…36.9]	24.5 [18.7…39.0]	23.0 [18.7…37.4]	31.1 [23.5…39.0]	0.360	0.021	0.426	0.007
**Pack years**	0.0 [0…0]	34.0 [10…60]	40.0 [15…60]	32.0 [10…50]	<0.001	<0.001	<0.001	0.284
**Medication** LABA without ICS/LABA plus ICS/LAMA	0 / 0 / 0	3 / 10 / 10	0 / 5 / 4	3 / 5 / 6	NA	NA	NA	NA
**FEV**_**1**_ (% pred.)	114.3 [93.9…126.6]	63.6 [41.5…80.0]	65.2 [41.5…80.0]	62.0 [51.5…71.4]	<0.001	<0.001	<0.001	0.392
**IVC** (% pred.)	107.1 [82.6…116.8]	86.7 [29.3…114.4]	83.6 [29.3…114.4]	88.8 [35.2…97.9]	0.008	0.115	0.002	0.608
**FEV**_**1 **_**% FVC** (%)	82.5 [77.2…98.3]	60.5 [34.4…71.5]	60.5 [34.4…71.5]	60.5 [47.4…66.8]	<0.001	<0.001	<0.001	0.955
**MEF50** (% pred.)	98.0 [72.2…156.2]	24.5 [11.7…39.4]	24.7 [11.7…39.4]	22.6 [16.9…32.0]	<0.001	<0.001	<0.001	0.649
**RV** (% pred.)	91.0 [79.6…136.0]	136.1 [87.1…185.4]	129.7 [115.3…185.4]	136.2 [87.1…183.8]	<0.001	<0.001	0.001	0.569
**TLC** (% pred.)	100.5 [85.8…121.5]	102.7 [86.1…148.9]	101.0 [89.3…148.9]	106.0 [86.1…127.0]	0.669	0.738	0.705	0.955
**Hemoglobin** (mmol/l)	9.2 [7.3…10.3]	9.1 [7.1…11.3]	9.3 [7.1…11.3]	8.9 [8.0…9.7]	0.809	0.832	0.468	0.531
**Hematocrit** (%)	43.0 [38.0…49.0]	44.0 [38…52]	45.0 [38.0…52.0]	41.0 [39.0…48.0]	0.897	0.605	0.756	0.691
**Platelets** (*10^9^/l)	229 [180…320]	236 [155…374]	246 [155…374]	226 [165…331]	0.985	0.693	0.705	0.608
**Leukocytes** (*10^9^/l)	5.9 [3.8…8.2]	7.5 [3.9…12.3]	7.7 [3.9…10.2]	7.3 [4.0…12.3]	0.101	0.077	0.314	0.955
**CRP** (mg/l)	1.1 [1.0…9.5]	2.1 [1.0…15.1]	1.9 [1.0…10.6]	2.3 [1.0…15.1]	0.170	0.446	0.099	0.459

Shown are general characteristics of the subjects, results of pulmonary function tests and blood parameters. All patients treated with inhaled corticosteroids (ICS) were also treated with a long-acting beta-agonist (LABA) in a fixed combination (“LABA plus ICS”). Parameters are displayed as median values [minimum…maximum]. Abbreviations denote: c-reactive protein (CRP), Inspiratory vital capacity (IVC), Forced expiratory volume in the first one second (FEV1), ratio of the FEV1 to the forced vital capacity (FEV1/FVC), maximum expiratory flow at 50% of VC (MEF50), residual volume (RV), total lung capacity (TLC). The four columns on the right side of the table show comparisons between the groups: Control vs. total group of COPD patients (C-T), Control vs. COPD (current smoker) (C-S), Control vs. COPD (former smoker) (C-F) and COPD (current smoker) vs. COPD (former smoker) (S-F).

### DC concentrations in peripheral blood and BALF

There was a non-significant trend to increased concentrations of pDCs in peripheral blood in patients with COPD, as compared to controls. Blood pDC concentrations were significantly higher in current smokers than in former smokers with COPD (Table 2). Blood mDC concentrations did not differ between the groups (Table 2). Compared to controls, pDCs (but not mDCs) in BALF were significantly increased in current smokers, but not former smokers with COPD (Table 2). Both mDC and pDC concentrations were significantly higher in BALF of current smokers than in former smokers with COPD (Table 2). Percentages of pDCs and mDCs in BALF did not significantly differ between the groups (Table 2).

**Table 2:** Dendritic cell concentrations and characteristics

	**Control group (C) n = 10**	**COPD total group (T) n = 24**	**COPD current smoker (S) n = 13**	**COPD former smoker (F) n = 11**	**C-T p**	**C-S p**	**C-F p**	**S-F p**
**Blood pDC** (*10^3^/ml)	0.64 [0.18…1.40]	0.80 [0.29…1.80]	0.95 [0.45…1.80]	0.68 [0.29…1.60]	0.564	0.284	0.918	0.013
**Blood mDC** (*10^3^/ml)	1.29 [0.49…1.82]	1.11 [0.54…2.46]	1.09 [0.54…2.14]	1.12 [0.68…2.46]	0.867	0.648	0.863	0.910
**BALF recovery** (ml)	55.0 [50.0…64.0]	45.5 [27.0…60.0]	47.0 [27.0…60.0]	45.0 [30.0…60.0]	0.007	0.026	0.016	0.608
**BALF cell count** (*10^3^/ml)	63.0 [21.0…106.0]	86.0 [6.0…530.0]	113.0 [45.0…530.0]	16.0 [6.0…259.0]	0.401	0.010	0.223	0.018
**BALF pDC** (*10^3^/ml)	0.03 [0.01…0.20]	0.06 [0.0…0.55]	0.07 [0.0…0.55]	0.01 [0.0…0.15]	0.564	0.042	0.251	0.035
**BALF pDC** (% of all cells)	0.055 [0.01…0.26]	0.07 [0.0…0.25]	0.07 [0.0…0.25]	0.06 [0.03…0.17]	0.956	0.784	0.863	0.910
**BALF mDC** (*10^3^/ml)	0.60 [0.20…1.62]	0.73 [0.06…4.13]	0.96 [0.21…4.13]	0.16 [0.06…1.68]	0.752	0.057	0.132	0.013
**BALF mDC** (% of all cells)	1.06 [0.53…1.53]	0.87 [0.32…2.76]	0.76 [0.32…2.76]	0.95 [0.35…1.71]	0.183	0.088	0.605	0.228
**BDCA-1+** (% mDC)	58.1 [44.0…69.0]	62.7 [23.0…93.0]	80.1 [52.9…93.0]	43.5 [23.0…64.6]	0.752	0.003	0.006	<0.001
**BDCA-3+** (% mDC)	58.8 [55.6…79.5]	57.7 [21.5…82.6]	61.7 [21.5…82.6]	56.8 [37.1…69.0]	0.148	0.313	0.132	0.608
**BDCA-4+** (% mDC)	9.5 [3.9…28.6]	26.2 [0.0…68.9]	43.3 [13.8…68.9]	8.1 [0.0…28.9]	0.038	<0.001	0.605	<0.001
**CD80+** (% mDC)	56.7 [45.6…73.5]	58.8 [30.0…79.1]	63.1 [44.2…79.1]	45.9 [30.0…60.9]	0.724	0.166	0.020	0.002
**CD83+** (% mDC)	38.4 [20.6…45.8]	28.4 [13.4…51.5]	23.7 [13.4.…39.0]	34.2 [21.2…51.5]	0.109	0.010	0.918	0.055
**CD86+** (% mDC)	80.3 [67.1…84.5]	85.2 [49.6…96.7]	89.3 [65.9…96.7]	78.0 [49.6…86.2]	0.118	0.004	0.863	0.002
**CCR5+** (% mDC)	27.9 [12.3…51.6]	11.1 [1.8…28.8]	10.9 [1.8…28.8]	12.9 [5.7…23.4]	0.001	0.002	0.008	0.865
**MMR +** (% mDC)	44.9 [30.6…56.1]	53.7 [22.8…84.0]	69.1 [50.6…84.0]	40.4 [22.8…69.2]	0.101	<0.001	0.251	<0.001
**Langerin +** (% mDC)	32.8 [19.7…51.0]	34.1 [9.4…76.8]	52.6 [28.3…76.8]	20.4 [9.4…44.6]	0.642	0.003	0.013	<0.001
**CD1a +** (% mDC)	43.5 [29.9…57.6]	56.3 [19.5…82.7]	72.7 [51.1…82.7]	35.6 [19.5…62.1]	0.196	<0.001	0.099	<0.001
**RANTES / CCL5** (pg/ml)	10.1 [4.0…66.5]	12.6 [4.0…500.0]	7.5 [4.0…500.0]	14.6 [4.0…49.4]	0.669	0.483	0.973	0.494

Parameters are displayed as median values [minimum…maximum]. Abbreviations denote: Bronchoalveolar Lavage Fluid (BALF), plamacytoid Dendritic Cell (pDC), myeloid Dendritic Cell (mDC), Blood Dendritic Cell Antigen (BDCA), Cluster of Differentiation (CD), Mannose Macrophage Receptor (MMR), Chemokine Receptor (CCR), Regulated on Activation, Normal T Cell Expressed and Secreted (RANTES, also Chemokine Ligand 5, CCL5). The four columns on the right side of the table show comparisons between the groups: Control vs. total group of COPD patients (C-T), Control vs. COPD (current smoker) (C-S), Control vs. COPD (former smoker) (C-F) and COPD (current smoker) vs. COPD (former smoker) (S-F).

### Surface molecule expression on BALF mDCs

**Figure 3:**
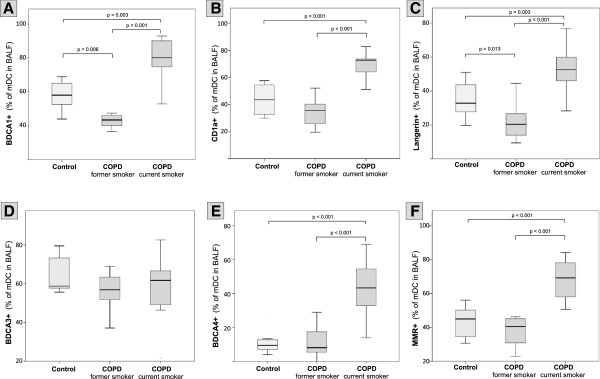
**Surface molecule expression on BALF mDCs.** Boxplots show the expression (% positive mDCs in BALF) of BDCA-1 **(A)**, CD1a **(B)**, Langerin **(C)**, BDCA-3 **(D)**, BDCA-4 **(E)** and MMR **(F)** on mDCs in BALF from healthy never-smokers (Control), former smokers with COPD (COPD, former smoker) and current smokers with COPD (COPD, current smoker). Boxplots display the median (line within the box), interquartil range (edges of the box) and extremes (vertical lines). Outliers (all cases more distant than 1.5 interquartil ranges from the upper or lower quartil) were omitted in the graphs. Significant differences between two time groups are marked with the exact p-value.

**Figure 4:**
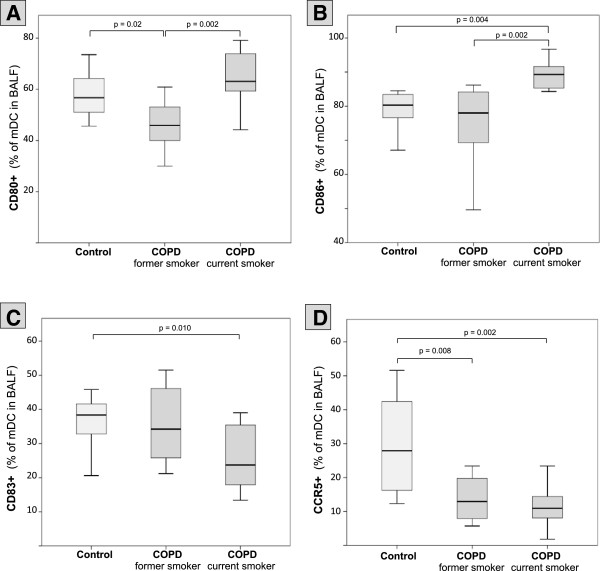
**Surface molecule expression on BALF mDCs.** Boxplots show the expression (% positive mDCs in BALF) of CD80 **(A)**, CD86 **(B)**, CD83 **(C)** and CCR5 **(D)** on mDCs in BALF from healthy never-smokers (Control), former smokers with COPD (COPD, former smoker) and current smokers with COPD (COPD, current smoker). Boxplots display the median (line within the box), interquartil range (edges of the box) and extremes (vertical lines). Outliers (all cases more distant than 1.5 interquartil ranges from the upper or lower quartil) were omitted in the graphs. Significant differences between two time groups are marked with the exact p-value.

Due to the low number of pDCs in BALF, a comprehensive and reliable flow cytometric analysis of surface-molecules on BALF pDCs could not be performed. Compared with controls, BALF mDCs of current smokers with COPD were characterized by an increased expression of CD1a, BDCA-1, Langerin, BDCA-4, MMR and CD86 (Figures 3, 4 and Table 2). In contrast, former smokers with COPD showed a significantly decreased expression of BDCA-1, Langerin and CD80, as compared with controls. Trends to a decreased expression of CD1a, MMR and CD86 on mDCs in former smokers were not significant (Figures 3, 4 and Table 2). Expression of BDCA-1, CD1a, Langerin, BDCA-4, MMR, CD80 and CD86 was significantly higher in current smokers than in former smokers with COPD (Figures 3, 4 and Table 2). Of note, the expression of BDCA-3 (CD141, Thrombomodulin) did not differ between the groups (Figure 3 and Table 2).

Current smokers with COPD were characterized by a significantly lower expression of the maturation marker CD83 on mDCs, as compared with controls. The trend to a reduced expression of CD83 on mDCs of former smokers was not significant (Figure 4 and Table 2). Compared with controls, CCR5 was strongly decreased on mDCs of current and former smokers (median: < 50% of controls). There was no difference in CCR5 expression between current and former smokers with COPD (Figure 4 and Table 2). BALF concentrations of CCL3 (MIP-1alpha) and CCL4 (MIP-1beta) were below detection limit in all samples (data not shown). CCL5 (RANTES) concentrations in BALF did not differ between groups (Table 2).

### DC phenotypes and airflow limitation

The expression of BDCA-1, CD1a, Langerin and CD80 on BALF mDCs correlated with the MEF_50_ (% predicted) in current smokers with COPD (Additional file 1: Figure S1). There were non-significant trends towards positive correlations of MEF_50_ (% predicted) with MMR expression on BALF mDCs (r = 0.49, p = 0.09), the number of BALF mDCs (r = 0.53, p = 0.06) and the total number of BALF cells (r = 0.55, p = 0.054) in current smokers with COPD. In former smokers with COPD and in controls, no correlations of MEF_50_ with the expression of cell surface molecules were detected. There were no correlations of mDC surface molecule expressions with the FEV_1_ in all groups, with the only exception of a significant correlation between BDCA-1 on mDCs and the FEV_1_ (% predicted; r = 0.69, p = 0.009).

### DC characteristics and ICS/LABA treatment

Patients with fixed ICS/LABA combination therapy displayed significantly lower concentrations of total cells (Additional file 1: Figure S2-A) as well as mDCs (Additional file 1: Figure S2-B) in BALF, as compared to those patients not receiving this combination therapy. BALF pDC cell concentrations did not significantly differ between these two groups (p = 0.108). There was no significant difference in the expression of CCR5 on BALF mDCs (Additional file 1: Figure S2-C) and in the concentration of the CCR5 ligand RANTES between these two groups (Additional file 1: Figure S2-D). There were no significant differences in the expression of other surface molecules on mDCs between the two groups (data not shown).

## Discussion

This is the first comprehensive analysis of the expression of function-associated surface molecules on airway DCs in COPD. Our study revealed that, compared with never-smoking controls, airway mDCs of current smokers with COPD display an increased expression of receptors for antigen recognition such as BDCA-1 or Langerin, whereas mDCs of former smokers with COPD display a decreased expression of these receptors. Only current smokers with COPD, but not former smokers with COPD, were characterized by a significantly reduced expression of the maturation marker CD83 on mDCs. In contrast, the expression of the chemokine receptor CCR5 was strongly reduced in all patients with COPD, independently of the smoking status.

The upregulation of surface molecules for antigen recognition and presentation (such as CD1a, BDCA-1, Langerin, MMR, BDCA-4 and CD86) and the unchanged expression of BDCA-3 on BALF mDCs found in the current study in smokers with COPD is strikingly similar to the previously reported changes on BALF mDCs of smokers with normal spirometry [14]. In contrast, BALF mDCs of former smokers with COPD displayed a significantly decreased expression of BDCA-1, Langerin and CD80 and a trend to a decreased expression of CD1a, MMR, BDCA-4 and CD86 on BALF mDCs, as compared with never-smoking controls. Thus, changes of these mDC surface molecules appear to be related to current smoking, rather than to the presence of airway obstruction. Of note, in current smokers with COPD, we found a positive correlation between the expression of these molecules on mDCs and the MEF_50,_ a marker of flow limitation in peripheral airways. Therefore, it might be hypothesized that an upregulation of these molecules can have protective functions in smoke-exposed airways. However, further studies are needed to elucidate the precise role of these receptors in smoke-related lung diseases.

DC maturation after antigen uptake is a crucial step for proper DC migration and antigen presentation to lymphocytes in the draining lymph nodes [23]. Smokers with normal spirometry display a normal expression of the maturation marker CD83 on BALF mDCs, suggesting that cigarette smoke does not per se affect airway DC maturation [14]. In immunohistochemical studies, Tsoumakidou and colleagues showed that smokers with COPD or asthma have decreased numbers of CD83 positive cells in the airways [12,26]. It remained unclear whether this observation represents a decrease in the total number of DCs or a reduced CD83 expression on DCs [27]. Here, we demonstrate that, in contrast to smokers with normal spirometry [14], smokers with COPD display a reduced CD83 expression on BALF mDCs. Thus, the data support the hypothesis by Tsoumakidou and colleagues that COPD might be associated with an impaired DC maturation, which could hamper DC migration to the draining lymph nodes [12].

CCR5 belongs to the family of CC chemokine receptors and plays a central role in the regulation of DC migration by binding to chemokines such as CCL3 (MIP-1alpha), CCL4 (MIP-1beta) and CCL5 (RANTES) [28]. In addition, CCR5 binds to various antigens derived from bacterial, fungal and viral pathogens. In some cases, a CCR5-mediated entry of these microbial antigens can have detrimental effects on immune cells and suppress antimicrobial immune responses [29,30]. For instance, the importance of CCR5 as the major human immunodeficiency virus (HIV) co-receptor controlling susceptibility to HIV infection led to the development of the CCR5 antagonist Maraviroc which has been approved by the FDA for the treatment of HIV infections [31]. However, a CCR5-mediated uptake of other microbial antigens by DCs can also stimulate antimicrobial immune responses, resulting in an improved clearance of these pathogens [32]. These data have been supported by human and experimental studies showing that heterozygous or homozygous carriers of dysfunctional CCR5 allels infected with the influenza A virus, the West nile virus or the tick-borne encephalitis virus suffer from a more severe clinical course of the disease [33-37]. Thus, it appears that a CCR5 deficiency can have protective or detrimental effects, depending on the specific infectious agent. We have previously shown that smokers with normal spirometry display a moderately decreased expression of the chemokine receptor CCR5 on BALF mDCs (mean values: 73% of controls) [14]. In the current study, it is demonstrated that COPD is associated with a strong decrease in the CCR5 expression of BALF mDCs (median values: < 50% of controls), independently of the smoking status. The significance of this CCR5 downregulation on mDCs in the airway lumen in COPD is currently unclear. Animal models of cigarette smoke-induced pulmonary inflammation suggest that CCR5 contributes to pulmonary inflammation and to the development of emphysema [38]. Another report showed a positive correlation between the expression of CCR5 on CD8+ T-cells in the airway wall and the severity of COPD [39]. It might be speculated that chronic elevation of chemokine concentrations in the airway wall of patients with COPD results in an accumulation of CCR5+ mDCs in the airway wall and a relative decrease of CCR5+ mDCs in the airway lumen. This reduced CCR5 expression on mDCs could hamper the interaction of mDCs with pathogens in the airway lumen. Therefore, further studies are needed to disentangle the precise role of this receptor in the immunopathology and microbiology of COPD [40].

Although the present study was neither intended nor powered to analyze the effects of medications on airway DCs in COPD, we performed a preliminary analysis comparing mDCs of patients treated with a fixed combination (ICS plus LABA) with those patients not treated with this therapy. This analysis suggests that treatment with a fixed combination is associated with reduced concentrations of airway mDCs in patients with COPD. Due to the limitations of the analysis (mainly the presence of other confounding factors and the small sample size of the subgroups), these results must be interpreted with caution. However, they should stimulate further research on the effects of ICS and LABA on immune cells in the airways of patients with COPD [41-44].

Our study has several limitations. First, only BALF DCs, but not DCs located within the airway wall were analyzed in this study. The phenotypes of the DCs in different compartments of the airway may differ substantially. Therefore, the phenotypes described in this report may not be representative for all airway compartments in patients with COPD. Second, due to technical limitations (there were too few pDCs in BALF for a reliable analysis of pDC surface molecules), we did not analyze the phenotype of plasmacytoid DCs (pDCs), although these cells may play an important role in the pathogenesis of COPD [45]. Third, the median BALF recovery was higher in the control group (median: 55 ml) than in the patients with COPD (median: 45 - 47 ml). This might have influenced total BALF DC counts in the groups. However, it appears unlikely that this difference affected the observed pattern of DC surface molecule expression. For instance, there was no significant in difference in BALF recovery between smokers and ex-smokers with COPD, but a strong difference in the expression of DC surface markers such as BDCA-1, CD1a and Langerin. Finally, the sample size in each group (controls, former smokers with COPD and current smokers with COPD) was relatively small. Although these sample sizes were sufficient to demonstrate the strong effects of smoking and the role of COPD regarding the expression of mDC surface molecules such as Langerin, BDCA-1 and CCR5, they did not allow for further subgroup analyses to identify possible confounding factors.

## Conclusion

This first comprehensive analysis of DC phenotypes in COPD adds important new information to the ongoing discussion on the role of DCs in the pathophysiology of COPD.

## Abbreviations

BALF: Bronchoalveolar Lavage Fluid; BDCA: Blood Dendritic Cell Antigen; CCR5: C-C Chemokine Receptor 5; CD: Cluster of Differentiation; COPD: Chronic Obstructive Pulmonary Disease; DC: Dendritic Cell; FACS: Fluorescence Activated Cell Sorter; FEV1: Forced expiratory volume in the first second; ICS: Inhaled corticosteroid; LABA: Long-Acting Beta-Agonist; LAMA: Long-Acting Muscarinic Antagonist; mDC: Myeloid Dendritic Cell; MEF50: Maximum expiratory flow when 50% of the forced vital capacity is exhaled; MMR: Macrophage Mannose Receptor; pDC: Plasmacytoid Dendritic Cell.

## Competing interests

PS, AH, AB, KG, MK, KB have no conflicts of interest. ML and JCV served in advisory boards and/or received lecture fees from the following companies: Astra Zeneca, Boehringer Ingelheim, Berlin-Chemie, Chiesi, GSK, Janssen, MSD, Novartis.

## Authors’ contributions

PS, JCV and ML designed and supervised the study, wrote the proposals for the authorities, analyzed the data and wrote the manuscript draft; PS and AH recruited and characterized the participants; PS, AB and ML performed the bronchoscopies; KG, MK and KB performed flow cytometric measurements and analyses. All authors read and approved the final manuscript.

## Supplementary Material

Additional file 1: Table S1 Antibodies used for four-colour flow cytometry. *Abbreviations denote:* Blood Dendritic Cell Antigen (BDCA), Fluorescein isothiocyanate (FITC), Phycoerythrin (PE), Allophycocyanin (APC), Peridinin chlorophyll protein (PerCP), Macrophage Mannose Receptor (MMR). **Figure S1.** DC surface molecules and airflow limitation in current smokers with COPD. *Abbreviations denote:* Blood Dendritic Cell Antigen (BDCA), Fluorescein isothiocyanate (FITC), Phycoerythrin (PE), Allophycocyanin (APC), Peridinin chlorophyll protein (PerCP), Macrophage Mannose Receptor (MMR). The figure shows the correlation between the expression of CD80 (A), BDCA-1 (B), CD1a (C) and Langerin (D) on BALF mDCs (% positive mDCs in BALF) and the maximum expiratory flow when 50% of the forced vital capacity is exhaled (MEF_50_, in % of the predicted value) in current smokers with COPD. The Spearman correlation coefficient (r) and the significance of the association (p) is given for each marker. **Figure S2.** Impact of fixed combination therapy on mDCs in BALF. Ten patients with COPD were treated with a fixed combination (+ICS/+LABA), whereas 14 patients did not receive this combination therapy (-ICS/-LABA). Boxplots show the total number of BALF cells (A), the total number of mDCs (B), the expression (% positive mDCs in BALF) of CCR5 on mDCs in BALF (C) and the concentration of the CCR5 ligand RANTES in BALF in both subgroups. Boxplots display the median (line within the box), interquartil range (edges of the box) and extremes (vertical lines). Outliers (all cases more distant than 1.5 interquartil ranges from the upper or lower quartil) were omitted in the graphs. Significant differences between two time groups are marked with the exact p-value. Click here for file

## References

[B1] Brusselle GG Joos GF Bracke KR New insights into the immunology of chronic obstructive pulmonary disease Lancet 2011 378 1015 1026 10.1016/S0140-6736(11)60988-4 21907865

[B2] Hogg JC Chu F Utokaparch S Woods R Elliott WM Buzatu L Cherniack RM Rogers RM Sciurba FC Coxson HO Pare PD The nature of small-airway obstruction in chronic obstructive pulmonary disease N Engl J Med 2004 350 2645 2653 10.1056/NEJMoa032158 15215480

[B3] Tsoumakidou M Demedts IK Brusselle GG Jeffery PK Dendritic cells in chronic obstructive pulmonary disease: new players in an old game Am J Respir Crit Care Med 2008 177 1180 1186 10.1164/rccm.200711-1727PP 18337593

[B4] Brusselle GG Demoor T Bracke KR Brandsma CA Timens W Lymphoid follicles in (very) severe COPD: beneficial or harmful? Eur Respir J 2009 34 219 230 10.1183/09031936.00150208 19567605

[B5] Cosio MG Saetta M Agusti A Immunologic aspects of chronic obstructive pulmonary disease N Engl J Med 2009 360 2445 2454 10.1056/NEJMra0804752 19494220

[B6] Tzortzaki EG Siafakas NM A hypothesis for the initiation of COPD Eur Respir J 2009 34 310 315 10.1183/09031936.00067008 19648516

[B7] Demedts IK Bracke KR Van Pottelberge G Testelmans D Verleden GM Vermassen FE Joos GF Brusselle GG Accumulation of dendritic cells and increased CCL20 levels in the airways of patients with chronic obstructive pulmonary disease Am J Respir Crit Care Med 2007 175 998 1005 10.1164/rccm.200608-1113OC 17332482

[B8] Van Pottelberge GR Bracke KR Demedts IK De Rijck K Reinartz SM van Drunen CM Verleden GM Vermassen FE Joos GF Brusselle GG Selective accumulation of langerhans-type dendritic cells in small airways of patients with COPD Respir Res 2010 11 35 10.1186/1465-9921-11-35 20307269 PMC2858735

[B9] Vassallo R Walters PR Lamont J Kottom TJ Yi ES Limper AH Cigarette smoke promotes dendritic cell accumulation in COPD; a Lung Tissue Research Consortium study Respir Res 2010 11 45 10.1186/1465-9921-11-45 20420706 PMC2867978

[B10] Mori M Andersson CK Svedberg KA Glader P Bergqvist A Shikhagaie M Lofdahl CG Erjefalt JS Appearance of remodelled and dendritic cell-rich alveolar-lymphoid interfaces provides a structural basis for increased alveolar antigen uptake in chronic obstructive pulmonary disease Thorax 2013 68 521 531 10.1136/thoraxjnl-2012-202879 23412435

[B11] Freeman CM Martinez FJ Han MK Ames TM Chensue SW Todt JC Arenberg DA Meldrum CA Getty C McCloskey L Curtis JL Lung dendritic cell expression of maturation molecules increases with worsening chronic obstructive pulmonary disease Am J Respir Crit Care Med 2009 180 1179 1188 10.1164/rccm.200904-0552OC 19729666 PMC2796731

[B12] Tsoumakidou M Koutsopoulos AV Tzanakis N Dambaki K Tzortzaki E Zakynthinos S Jeffery PK Siafakas NM Decreased small airway and alveolar CD83+ dendritic cells in COPD Chest 2009 136 726 733 10.1378/chest.08-2824 19465512

[B13] Bratke K Lommatzsch M Julius P Kuepper M Kleine HD Luttmann W Christian Virchow J Dendritic cell subsets in human bronchoalveolar lavage fluid after segmental allergen challenge Thorax 2007 62 168 175 10.1136/thx.2006.067793 16928719 PMC2111237

[B14] Bratke K Klug M Bier A Julius P Kuepper M Virchow JC Lommatzsch M Function-associated surface molecules on airway dendritic cells in cigarette smokers Am J Respir Cell Mol Biol 2008 38 655 660 10.1165/rcmb.2007-0400OC 18203971

[B15] Lommatzsch M Bratke K Bier A Julius P Kuepper M Luttmann W Virchow JC Airway dendritic cell phenotypes in inflammatory diseases of the human lung Eur Respir J 2007 30 878 886 10.1183/09031936.00036307 17626112

[B16] Lommatzsch M Bratke K Knappe T Bier A Dreschler K Kuepper M Stoll P Julius P Virchow JC Acute effects of tobacco smoke on human airway dendritic cells in vivo Eur Respir J 2010 35 1130 1136 10.1183/09031936.00090109 19741025

[B17] Aquino A Graziani G Franzese O Prete SP Bonmassar E Bonmassar L D'Atri S Exogenous control of the expression of Group I CD1 molecules competent for presentation of microbial nonpeptide antigens to human T lymphocytes Clin Dev Immunol 2011 2011 790460 21603161 10.1155/2011/790460 PMC3095450

[B18] van der Vlist M Geijtenbeek TB Langerin functions as an antiviral receptor on Langerhans cells Immunol Cell Biol 2010 88 410 415 10.1038/icb.2010.32 20309013

[B19] Martinez-Pomares L The mannose receptor J Leukoc Biol 2012 92 1177 1186 10.1189/jlb.0512231 22966131

[B20] Tordjman R Lepelletier Y Lemarchandel V Cambot M Gaulard P Hermine O Romeo PH A neuronal receptor, neuropilin-1, is essential for the initiation of the primary immune response Nat Immunol 2002 3 477 482 11953749 10.1038/ni789

[B21] Bakdash G Sittig SP van Dijk T Figdor CG de Vries IJ The nature of activatory and tolerogenic dendritic cell-derived signal II Front Immunol 2013 4 53 23450201 10.3389/fimmu.2013.00053 PMC3584294

[B22] Morser J Thrombomodulin links coagulation to inflammation and immunity Curr Drug Targets 2012 13 421 431 10.2174/138945012799424606 22206250

[B23] Prechtel AT Steinkasserer A CD83: an update on functions and prospects of the maturation marker of dendritic cells Arch Dermatol Res 2007 299 59 69 10.1007/s00403-007-0743-z 17334966

[B24] Dreschler K Bratke K Petermann S Bier A Thamm P Kuepper M Virchow JC Lommatzsch M Impact of immunotherapy on blood dendritic cells in patients with Hymenoptera venom allergy J Allergy Clin Immunol 2011 127 487 494 e481-483 10.1016/j.jaci.2010.12.003 21281873

[B25] Segura E Touzot M Bohineust A Cappuccio A Chiocchia G Hosmalin A Dalod M Soumelis V Amigorena S Human inflammatory dendritic cells induce Th17 cell differentiation Immunity 2013 38 336 348 10.1016/j.immuni.2012.10.018 23352235

[B26] Tsoumakidou M Elston W Zhu J Wang Z Gamble E Siafakas NM Barnes NC Jeffery PK Cigarette smoking alters bronchial mucosal immunity in asthma Am J Respir Crit Care Med 2007 175 919 925 10.1164/rccm.200607-908OC 17303795

[B27] Tsoumakidou M Jeffery PK Dendritic cell maturity and obstructive airway disease Am J Respir Crit Care Med 2007 176 833 author reply 833-834. 10.1164/ajrccm.176.8.833a 17914125

[B28] Bachelerie F Ben-Baruch A Burkhardt AM Combadiere C Farber JM Graham GJ Horuk R Sparre-Ulrich AH Locati M Luster AD Mantovani A Matsushima K Murphy PM Nibbs R Nomiyama H Power CA Proudfoot AE Rosenkilde MM Rot A Sozzani S Thelen M Yoshie O Zlotnik A International Union of Pharmacology. LXXXIX. Update on the extended family of chemokine receptors and introducing a new nomenclature for atypical chemokine receptors Pharmacol Rev 2014 66 1 79 24218476 10.1124/pr.113.007724 PMC3880466

[B29] Alonzo F 3rd Kozhaya L Rawlings SA Reyes-Robles T DuMont AL Myszka DG Landau NR Unutmaz D Torres VJ CCR5 is a receptor for Staphylococcus aureus leukotoxin ED Nature 2013 493 51 55 23235831 10.1038/nature11724 PMC3536884

[B30] Cameron PU Handley AJ Baylis DC Solomon AE Bernard N Purcell DF Lewin SR Preferential infection of dendritic cells during human immunodeficiency virus type 1 infection of blood leukocytes J Virol 2007 81 2297 2306 10.1128/JVI.01795-06 17166903 PMC1865918

[B31] Henrich TJ Kuritzkes DR HIV-1 entry inhibitors: recent development and clinical use Curr Opin Virol 2013 3 51 57 10.1016/j.coviro.2012.12.002 23290628 PMC4213740

[B32] Floto RA MacAry PA Boname JM Mien TS Kampmann B Hair JR Huey OS Houben EN Pieters J Day C Oehlmann W Singh M Smith KG Lehner PJ Dendritic cell stimulation by mycobacterial Hsp70 is mediated through CCR5 Science 2006 314 454 458 10.1126/science.1133515 17053144

[B33] Dawson TC Beck MA Kuziel WA Henderson F Maeda N Contrasting effects of CCR5 and CCR2 deficiency in the pulmonary inflammatory response to influenza A virus Am J Pathol 2000 156 1951 1959 10.1016/S0002-9440(10)65068-7 10854218 PMC1850091

[B34] Glass WG McDermott DH Lim JK Lekhong S Yu SF Frank WA Pape J Cheshier RC Murphy PM CCR5 deficiency increases risk of symptomatic West Nile virus infection J Exp Med 2006 203 35 40 10.1084/jem.20051970 16418398 PMC2118086

[B35] Kindberg E Mickiene A Ax C Akerlind B Vene S Lindquist L Lundkvist A Svensson L A deletion in the chemokine receptor 5 (CCR5) gene is associated with tickborne encephalitis J Infect Dis 2008 197 266 269 10.1086/524709 18179389

[B36] Keynan Y Juno J Meyers A Ball TB Kumar A Rubinstein E Fowke KR Chemokine receptor 5 Δ32 allele in patients with severe pandemic (H1N1) 2009 Emerg Infect Dis 2010 16 1621 1622 10.3201/eid1610.100108 20875295 PMC3294998

[B37] Rodriguez A Falcon A Cuevas MT Pozo F Guerra S Garcia-Barreno B Martinez-Orellana P Perez-Brena P Montoya M Melero JA Pizarro M Ortin J Casas I Nieto A Characterization in vitro and in vivo of a pandemic H1N1 influenza virus from a fatal case PLoS One 2013 8 e53515 10.1371/journal.pone.0053515 23326447 PMC3542358

[B38] Bracke KR D'Hulst AI Maes T Demedts IK Moerloose KB Kuziel WA Joos GF Brusselle GG Cigarette smoke-induced pulmonary inflammation, but not airway remodelling, is attenuated in chemokine receptor 5-deficient mice Clin Exp Allergy 2007 37 1467 1479 17883726 10.1111/j.1365-2222.2007.02808.x

[B39] Freeman CM Curtis JL Chensue SW CC chemokine receptor 5 and CXC chemokine receptor 6 expression by lung CD8+ cells correlates with chronic obstructive pulmonary disease severity Am J Pathol 2007 171 767 776 10.2353/ajpath.2007.061177 17640964 PMC1959492

[B40] Bracke KR Demedts IK Joos GF Brusselle GG CC-chemokine receptors in chronic obstructive pulmonary disease Inflamm Allergy Drug Targets 2007 6 75 79 10.2174/187152807780832292 17692030

[B41] Barnes NC Qiu YS Pavord ID Parker D Davis PA Zhu J Johnson M Thomson NC Jeffery PK Antiinflammatory effects of salmeterol/fluticasone propionate in chronic obstructive lung disease Am J Respir Crit Care Med 2006 173 736 743 10.1164/rccm.200508-1321OC 16424444

[B42] Bourbeau J Christodoulopoulos P Maltais F Yamauchi Y Olivenstein R Hamid Q Effect of salmeterol/fluticasone propionate on airway inflammation in COPD: a randomised controlled trial Thorax 2007 62 938 943 10.1136/thx.2006.071068 17557771 PMC2117108

[B43] Rossios C To Y Osoata G Ito M Barnes PJ Ito K Corticosteroid insensitivity is reversed by formoterol via phosphoinositide-3-kinase inhibition Br J Pharmacol 2012 167 775 786 10.1111/j.1476-5381.2012.01864.x 22251095 PMC3575778

[B44] Lommatzsch M Kraeft U Troebs L Garbe K Bier A Stoll P Klammt S Kuepper M Bratke K Virchow JC Fluticasone impact on airway dendritic cells in smokers: a randomized controlled trial Respir Res 2013 14 114 10.1186/1465-9921-14-114 24168756 PMC4176093

[B45] Van Pottelberge GR Bracke KR Van den Broeck S Reinartz SM van Drunen CM Wouters EF Verleden GM Vermassen FE Joos GF Brusselle GG Plasmacytoid dendritic cells in pulmonary lymphoid follicles of patients with COPD Eur Respir J 2010 36 781 791 10.1183/09031936.00140409 20351031

