# Supplementary material for: Impact of smoking on dendritic cell phenotypes in the airway lumen of patients with COPD
Source: Respir Res. 2014 Apr 18;15(1):48. doi: 10.1186/1465-9921-15-48 (PMC4021430; doi:10.1186/1465-9921-15-48)
Supplement: Additional file 1: Table S1 — Antibodies used for four-colour flow cytometry. Abbreviations denote: Blood Dendritic Cell Antigen (BDCA), Fluorescein isothiocyanate (FITC), Phycoerythrin (PE), Allophycocyanin (APC), Peridinin chlorophyll protein (PerCP), Macrophage Mannose Receptor (MMR). Figure S1. DC surface molecules and airflow limitation in current smokers with COPD. Abbreviations denote: Blood Dendritic Cell Antigen (BDCA), Fluorescein isothiocyanate (FITC), Phycoerythrin (PE), Allophycocyanin (APC), Peridinin chlorophyll protein (PerCP), Macrophage Mannose Receptor (MMR). The figure shows the correlation between the expression of CD80 (A), BDCA-1 (B), CD1a (C) and Langerin (D) on BALF mDCs (% positive mDCs in BALF) and the maximum expiratory flow when 50% of the forced vital capacity is exhaled (MEF50, in % of the predicted value) in current smokers with COPD. The Spearman correlation coefficient (r) and the significance of the association (p) is given for each marker. Figure S2. Impact of fixed combination therapy on mDCs in BALF. Ten patients with COPD were treated with a fixed combination (+ICS/+LABA), whereas 14 patients did not receive this combination therapy (-ICS/-LABA). Boxplots show the total number of BALF cells (A), the total number of mDCs (B), the expression (% positive mDCs in BALF) of CCR5 on mDCs in BALF (C) and the concentration of the CCR5 ligand RANTES in BALF in both subgroups. Boxplots display the median (line within the box), interquartil range (edges of the box) and extremes (vertical lines). Outliers (all cases more distant than 1.5 interquartil ranges from the upper or lower quartil) were omitted in the graphs. Significant differences between two time groups are marked with the exact p-value. [file 1465-9921-15-48-S1.doc]

**Impact of smoking on dendritic cell phenotypes**

**in the airway lumen of patients with COPD**

Paul Stoll, MD^1^, Ann-Sophie Heinz^1^, Kai Bratke, PhD^1^, Andrea Bier, MD^1^,

Katharina Garbe^1^, PhD, Michael Kuepper, PhD^1^, J. Christian Virchow, MD^1^

and Marek Lommatzsch, MD^1§^

^1^Department of Pneumology and Critical Care Medicine, University of Rostock, Germany

# Additional file 1

| **Antigen** | **Label** | **Clone** | **Company** |
| --- | --- | --- | --- |
|  | | | |
| **Lineage cocktail** | | | |
| CD3 | FITC | SK7 | BD Biosciences |
| CD14 | FITC | MφP9 | BD Biosciences |
| CD16 | FITC | 3G8 | BD Biosciences |
| CD19 | FITC | SJ25C1 | BD Biosciences |
| CD20 | FITC | L27 | BD Biosciences |
| CD56 | FITC | NCAM16.2 | BD Biosciences |
|  | | | |
| **Other antibodies** | | | |
| CD1a | PE | NA1/34 | Dako |
| CD11c | PE | S-HCL-3 | BD Biosciences |
| CD123 | PE | 9F5 | BD Biosciences |
| Langerin | PE | DCGM4 | Beckmann Coulter |
| HLA-DR | PerCP | L243 | BD Biosciences |
| BDCA-1 | APC | AD5-8E7 | Miltenyi Biotec |
| BDCA-3 | APC | AD5-14H12 | Miltenyi Biotec |
| BDCA-4 | APC | AD5-17F6 | Miltenyi Biotec |
| CD11c | APC | S-HCL-3 | BD Biosciences |
| CD80 | APC | MEM-233 | ImmunoTools |
| CD83 | APC | HB15e | Invitrogen |
| CD86 | APC | BU63 | Invitrogen |
| CCR5 | APC | 2D7 | BD Biosciences |
| MMR | APC | 19.2 | BD Biosciences |

**Table S1. Antibodies used for four-colour flow cytometry**

*Abbreviations denote:* Blood Dendritic Cell Antigen (BDCA), Fluorescein isothiocyanate (FITC), Phycoerythrin (PE), Allophycocyanin (APC), Peridinin chlorophyll protein (PerCP), Macrophage Mannose Receptor (MMR).

#
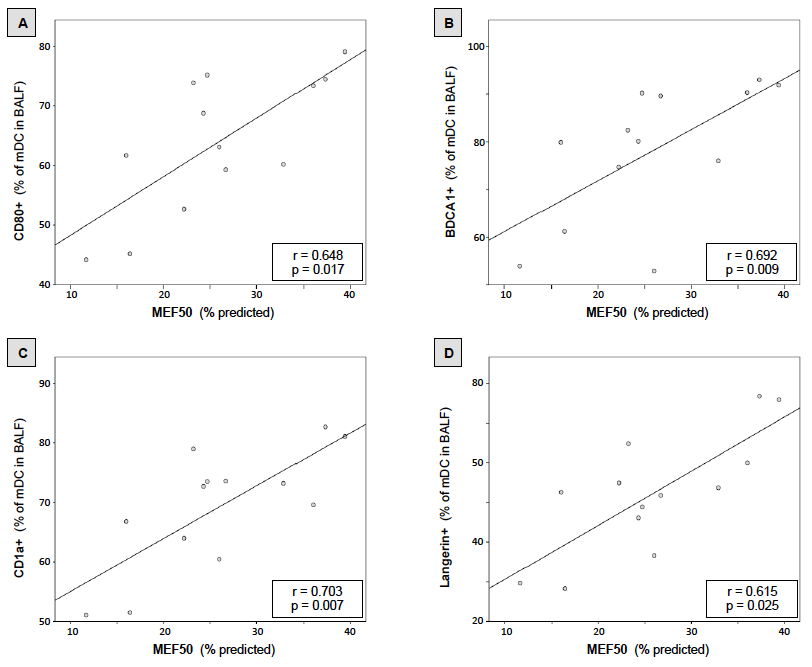


**Figure S1. DC surface molecules and airflow limitation in current smokers with COPD**

The figure shows the correlation between the expression of CD80 (**A**), BDCA-1 (**B**), CD1a (**C**) and Langerin (**D**) on BALF mDCs (% positive mDCs in BALF) and the maximum expiratory flow when 50% of the forced vital capacity is exhaled (MEF_50_, in % of the predicted value) in current smokers with COPD. The Spearman correlation coefficient (r) and the significance of the association (p) is given for each marker.

#
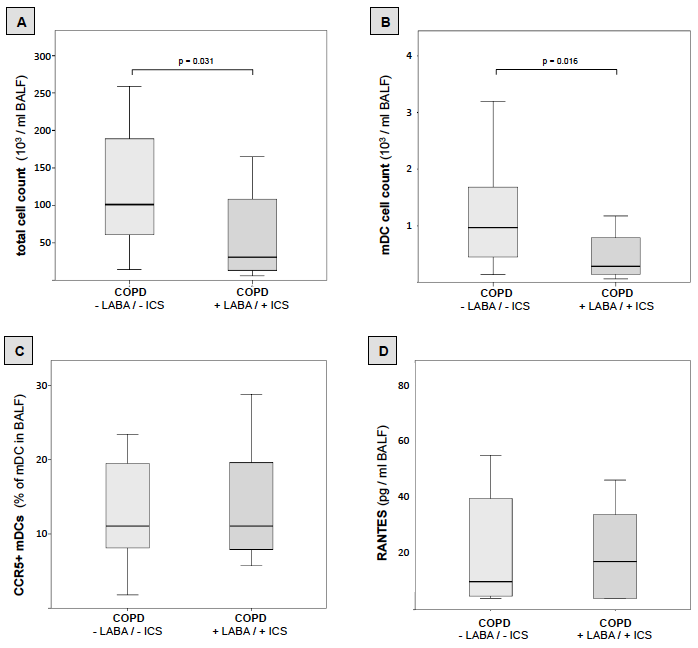


**Fig. S2. Impact of fixed combination therapy on mDCs in BALF**

Ten patients with COPD were treated with a fixed combination **(+ICS/+LABA**), whereas 14 patients did not receive this combination therapy **(-ICS/-LABA**). Boxplots show the total number of BALF cells (**A**), the total number of mDCs (**B**), the expression (% positive mDCs in BALF) of CCR5 on mDCs in BALF (**C**) and the concentration of the CCR5 ligand RANTES in BALF in both subgroups. Boxplots display the median (line within the box), interquartil range (edges of the box) and extremes (vertical lines). Outliers (all cases more distant than 1.5 interquartil ranges from the upper or lower quartil) were omitted in the graphs. Significant differences between two time groups are marked with the exact p-value.
